# Microglial inflammasome activation drives developmental white matter injury

**DOI:** 10.1002/glia.23963

**Published:** 2021-01-08

**Authors:** Rebecca K. Holloway, Graeme Ireland, Gemma Sullivan, Julie‐Clare Becher, Colin Smith, James P. Boardman, Pierre Gressens, Veronique E. Miron

**Affiliations:** ^1^ Medical Research Council Centre for Reproductive Health, The Queen's Medical Research Institute, The University of Edinburgh Edinburgh UK; ^2^ Simpson Centre for Reproductive Health, Royal Infirmary of Edinburgh Edinburgh UK; ^3^ Centre for Clinical Brain Sciences, Centre for Comparative Pathology, Chancellor's Building, The University of Edinburgh Edinburgh UK; ^4^ Department of Perinatal Imaging and Health Rayne's Institute, King's College London London UK; ^5^ PROTECT, INSERM, Université Paris Diderot, Sorbonne Paris Cité Paris France; ^6^ Present address: Aquila Biomedical, 9 Edinburgh Bioquarter Edinburgh UK

**Keywords:** inflammation, microglia, myelination, oligodendrocyte, white matter injury

## Abstract

Injury to the developing brain during the perinatal period often causes hypomyelination, leading to clinical deficits for which there is an unmet therapeutic need. Dysregulation of inflammation and microglia have been implicated, yet the molecular mechanisms linking these to hypomyelination are unclear. Using human infant cerebrospinal fluid (CSF) and postmortem tissue, we found that microglial activation of the pro‐inflammatory molecular complex the NLRP3 inflammasome is associated with pathology. By developing a novel mouse brain explant model of microglial inflammasome activation, we demonstrate that blocking the inflammasome rescues myelination. In human and mouse, we discovered a link between the inflammasome product IL1β and increased levels of follistatin, an endogenous inhibitor of activin‐A. Follistatin treatment was sufficient to reduce myelination, whereas myelination was rescued in injured explants upon follistatin neutralization or supplementation with exogenous activin‐A. Our data reveal that inflammasome activation in microglia drives hypomyelination and identifies novel therapeutic strategies to reinstate myelination following developmental injury.

## Main points

- Microglia inflammasome activation correlates with white matter injury.
- Inflammasome inhibition promotes myelination.
- IL1β increases the activin‐A inhibitor follistatin, which impedes myelination.
- Activin‐A enhances myelination following injury.

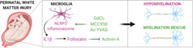

## 1 INTRODUCTION

Injury to the infant brain during the perinatal period is the predominant risk factor for development of lifelong deficits in cognition, sensation, or movement (e.g., as seen in cerebral palsy). Common contributors to this injury are infection‐induced inflammation and hypoxia, which alter oligodendrocyte precursor responses, including increased proliferation and death, and impaired maturation into myelin‐forming oligodendrocytes (Back & Rosenberg, 2014; Hagberg et al., 2015; van Tilborg et al., 2018). The consequent hypomyelination then deprives axons of metabolic/trophic support and insulation for electrical impulse conduction (Back et al., 2001; French, Reid, Mamontov, Simmons, & Grinspan, 2009; Segovia et al., 2008).

This injury is associated with dysregulation of resident macrophages of the central nervous system (CNS) termed microglia (Dommergues, Plaisant, Verney, & Gressens, 2003; Favrais et al., 2011; Hellstrom Erkenstam et al., 2016; Krishnan et al., 2017; Pang, Cai, & Rhodes, 2003; Verney et al., 2012), which show sustained expression of pro‐inflammatory factors (e.g., inducible nitric oxide synthase [iNOS] and tumor necrosis factor alpha [TNFα+]) (Haynes, Folkerth, Trachtenberg, Volpe, & Kinney, 2009; Pang et al., 2003; Yoon et al., 1997). However, the molecular mechanisms linking microglial dysregulation to hypomyelination remain unclear.

Here, we asked whether microglia cause hypomyelination in developing brain via activation of the pro‐inflammatory molecular complex the NLRP3 inflammasome, which has been previously linked to both dysregulated microglia activation and white matter injury in the adult CNS (Barclay & Shinohara, 2017; Denker et al., 2007; Inoue, Williams, Gunn, & Shinohara, 2012). We demonstrate microglial activation of the NLRP3 inflammasome to be a key pathological driver of developmental hypomyelination. We also reveal a hitherto unrecognized link between microglial inflammasome activation and regulation of activin‐A signaling, which limits developmental myelination after injury. Together, our findings identify novel therapeutic strategies to restore myelination following perinatal brain injury.

## 2 MATERIALS AND METHODS

### 2.1 Animal husbandry

All experiments were performed under V.E.M.'s United Kingdom Home Office project license issued under the “Animals (scientific procedures) Act 1986” and with approval of the Animal Welfare and Ethics Review Body. Animals were housed in a 12 hr light/dark cycle with unrestricted access to food and water. ARRIVE guidelines have been followed for this study.

### 2.2 Organotypic mouse brain explant model

Postnatal day (P)0‐P3 CD1 forebrains of male and female pups were coronally sectioned at 300 μm using a McIlwain tissue chopper and plated onto poly‐D‐lysine and laminin‐2‐coated (10 μg/ml) Millipore Millicel‐CM mesh inserts (Fisher Scientific) at three explants per insert. Media was composed of 25% Earle's Balanced Salt Solution (Life Technologies), 67% Basal Medium Eagle (Life Technologies), 27 mM glucose (Sigma‐Aldrich), 5% heat inactivated horse serum (Gibco), 1% penicillin/streptomycin and Glutamax (Life Technologies). Cultures were maintained at 37°C and 5% CO_2_ and media was changed every 2–3 days. At 7 days in vitro (DIV), inflammasome activation was induced in explants first by exposure to 100 ng/ml lipopolysaccharide from *Escherichia coli* (LPS; 0127:B8; Sigma) for 2 hr, then following washes with media, explants were cultured in fresh media in hypoxic conditions (3% O_2_) for 3 days until 10 DIV. In a subset of experiments, explants were treated with GdCl_3_ (270 μM, Sigma), MCC950 (10 μM, Selleck Chemicals), Ac‐YVAD (100 μM, Cambridge Bioscience), activin‐A (10 ng/ml, R&D systems) or vehicle controls during LPS and hypoxia exposure from 7 to 10 DIV. In a subset of experiments, a follistatin neutralizing antibody (10 μg/ml; R&D Systems) or goat IgG isotype control (10 μg/ml; R&D Systems) was applied during LPS and hypoxia exposure from 7 to 10 DIV, or recombinant follistatin (10 ng/ml; R&D Systems) or vehicle control was applied to uninjured explants from 7 to 10 DIV. Explants were fixed with 4% paraformaldehyde (PFA; Sigma) for 10 min.

### 2.3 Immunofluorescent staining of explants

Fixed explants were blocked in 5% normal horse serum (GIBCO) and 0.3% Triton‐X‐100 (Fisher Scientific) for 1 hr. Primary antibodies diluted in block were applied in a humid chamber at 4°C for 48 hr. Antibodies included mouse anti‐O4 (1:100, gift from J. Pound, University of Edinburgh), rat anti‐MBP (1:250, AbD Serotec), rabbit anti‐Ki67 (1:100, EMD Millipore), mouse anti‐Olig2 (1:100, EMD Millipore), rabbit anti‐Olig2 (1:100, EMD Millipore), mouse anti‐Olig1 (1:100, Millipore), mouse anti‐NFH (1:1000, EnCor), mouse anti‐iNOS (1:100, BD Biosciences), rabbit anti‐NG2 (1:200, EMD Millipore), goat anti‐cleaved caspase‐1 (p20/m296, 1:100, Santa Cruz Biotechnology), and rat anti‐CD68 (1:100, Abcam). Following washes in PBS, secondary antibody diluted in block was applied in a humid chamber at 20–25°C for 2 hr (1:500; Life Technologies‐Molecular Probes). To assess cell death, the DeadEND fluorometric TUNEL system (Promega) was used according to the manufacturer's instructions. Death was further assessed in explants by live incorporation of a marker of compromised membrane integrity, propidium iodide (PI, 25 μg/ml; Sigma‐Aldrich), for 1 hr prior to fixation in the dark. Explants were counterstained with Hoechst and mounted onto glass slides using Fluoromount‐G (Southern Biotech). Images were captured as Z‐stacks using an Olympus 3i Spinning Disk microscope and presented as maximum projections using SlideBook 6 software.

### 2.4 Enzyme‐linked immunoabsorbant assay

Levels of TNF‐α or follistatin in conditioned media collected from explants were measured by ELISA according to the manufacturer's instructions (R&D Systems; RayBiotech). Absorbance was read at 450 nm on a spectrophotometer and sample concentrations calculated using the equation generated from the standard curve.

### 2.5 Western blotting

Explants were washed in ice‐cold PBS, scraped off the mesh into RIPA buffer (Sigma‐Aldrich) supplemented with Thermo Scientific Halt protease and phosphatase inhibitor cocktail, and homogenized with a 23‐gauge needle. Samples were shaken at 4°C for 30 min, then centrifuged at 13,000 rpm for 10 min at 4°C. Samples were stored at −80°C until use. Protein concentrations were determined using the BCA Protein Assay Kit (Pierce) according to the manufacturer's instructions. Samples were diluted in Laemmli buffer (Bio‐Rad), supplemented with 5% mercapto‐ethanol, and heated at 95°C for 2 minutes. Five microgram of protein per sample was loaded onto 4–15% pre‐cast polyacrylamide gels (Bio‐Rad). Gel electrophoresis was performed in Tris‐hydroxyethyl piperazineethanesulfonic acid (HEPES)‐sodium dodecyl sulfate (SDS) running buffer (Thermo Scientific) at 100 V for 45 min. Proteins were transferred onto polyvinylidene difluoride (PVDF) membranes (Millipore) for 2 hr at 10 V in 10% Pierce transfer buffer (Thermo Scientific) and 20% methanol diluted in water. Membranes were blocked with 4% bovine serum albumin (BSA) in Tris‐buffered saline with 0.1% Tween‐20 (vol/vol) (TBST) for 1 hr at room temperature on an orbital shaker. Rabbit anti‐IL1β (1:1000, Abcam) was applied overnight at 4°C, washed thrice in TBST for 10 min and incubated with horseradish peroxidase‐conjugated secondary antibody (1:2000; Cell Signalling Technology) for 1 hr at room temperature. Chemiluminescent substrate detection reagent ECL SuperSignal (Thermo Scientific) was applied and digital imaging was performed using the LI‐COR Odyssey Fc system. For loading control, membranes were re‐blotted with mouse anti‐β‐actin antibody (1:2000, Cell Signalling Technology) then secondary antibody, and imaged as above.

### 2.6 Data mining of in vivo mouse model of perinatal brain injury

A microarray gene expression dataset of microglia isolated from a perinatal brain injury model was mined for expression of follistatin (*Fst*) (Krishnan et al., 2017). This data was generated following intraperitoneal injections of recombinant mouse interleukin‐1β (10 μg/kg) or PBS vehicle control into male Swiss mice (OF1) twice a day from P1‐4 and once at P5. Four hours postinjection, mice were sacrificed by perfusion with 0.9% sodium chloride. Mice were randomly allocated to treatment groups by alternating allocation. Brains were dissociated using the Neural Tissue Dissociation Kit containing papain and the gentle MACS Octo Dissociator with Heaters (Miltenyi). Microglia were isolated by magnetic bead associated cell sorting using the CD11B antibody (1:10; Miltenyi) according to the manufacturer's instructions. RNA was extracted and hybridized to Agilent Whole Genome Oligo Microarray (8x60K). Background corrected Log_2_ intensity data were quantile‐normalized and assigned *p* values based on intensity levels and proximity to baseline, with *p* < .05 taken to be statistically significant. Mean expression values from PBS controls were subtracted from the IL‐1β value at the respective time point. In the published study, the authors confirmed that the cells isolated for gene expression were microglia by flow cytometry (CD11b^+^ CD45^lo^ F480^hi^ Ly6G^−^) and were not neutrophils (Ly6G^+^) or monocytes (CD45^hi^). Furthermore, qPCR analysis of isolated cells found no expression of genes associated with oligodendrocytes, astrocytes, or neurons (Krishnan et al., 2017).

### 2.7 Human post‐mortem tissue, immunostaining, and image acquisition

All tissue was collected with consent and approved by institutional review boards. Paraffin‐embedded tissue was deparaffinized in Histoclear (twice for 10 min) and decreasing concentrations of ethanol (100–50%; 5 min each), then washed in 0.1% Tween20 (vol/vol) in Tris‐Buffered Saline (TBS). Slides were microwaved in Vector Unmasking Solution for 10 min, washed once and endogenous phosphatase and peroxidase activity blocked for 5 min (Bloxall, Vector). Primary antibody diluted in 2.5% Normal Horse Serum (Vector) was applied overnight in a humid chamber at 4°C. Antibodies used included rat anti‐myelin basic protein (MBP; 1:250, AbD Serotec), mouse anti‐CD68 (1:100, DAKO), rabbit anti‐iNOS (1:100, Novus), rabbit anti‐NLRP3 (1:100, Novus Biochemicals), and rabbit anti‐ENPP6 (1:100, ThermoFisher). Following washes, alkaline phosphatase‐conjugated secondary antibody was applied for 30 min at 20–25°C in a humid chamber. Sections were washed in TBS and visualized by Vector Blue substrate kit according to the manufacturer's instructions (maximum 15 min). For co‐staining, sections were washed thrice and re‐blocked to quench residual phosphatase activity (Bloxall, Vector) prior to application of primary antibody, then developed using Vector Red substrate kit according to the manufacturer's instructions (maximum 15 min). Following washes in water, the sections were counterstained with Hoechst and mounted with Fluoromount‐G. Entire tissue sections were imaged using a Zeiss AxioScan SlideScanner. Comparisons were made either to healthy control cases, or between injury and noninjury regions within the same section. Regions of interest were defined for analysis using Zeiss Zen2 software and representative fields of 360 μm × 360 μm were counted, and counts were multiplied to determine density of immunopositive cells per mm^2^.

### 2.8 Human infant cerebrospinal fluid analysis

The neonatal cohort used in this study has been previously described (Boardman et al., 2018). Sampling was carried out in accordance with the recommendations of UK National Research Ethics Service and South East Scotland Research Ethics Committee (14/SS/044) with written consent from parents of all subjects. We selected 11 preterm infants and 15 term neonates with a C‐Reactive Protein level of >1 mg/L, previously used as a proxy for subclinical inflammation (Fond, Lancon, Auquier, & Boyer, 2018). Infants with CRP >1 mg/L had reduced levels of proteins associated with healthy myelination (IGF1: 0.79 fold; SPP1: 0.64 fold; GAL3: 0.17 fold) suggestive of white matter pathology. For preterm versus term infants, the mean birthweights were 1,026 and 3,473 g, the percentage of males in each cohort was 72 and 80%, and the mean age at birth was 27.24 and 39.96 gestational weeks, respectively. No infants in either group had meningitis. We analyzed data previously generated from a custom antibody array (Tebu‐Bio/Ray Biotech) as described previously (Boardman et al., 2018) and used data post‐background normalization.

### 2.9 Statistics

Volocity 6.3 software (Perkin Elmer) was used to calculate area of co‐localization between MBP and neurofilament; these values were normalized to total area of neurofilament to account for variation in axonal densities (“myelination index”). Western blots were quantified using Image Studio Lite v5.2 software. All manual counts were carried out in a blinded manner. Mean ± SEM are shown in graphs. Data handling and statistical processing performed using Microsoft Excel and GraphPad Prism 8 software.

## 3 RESULTS

**FIGURE 1:**
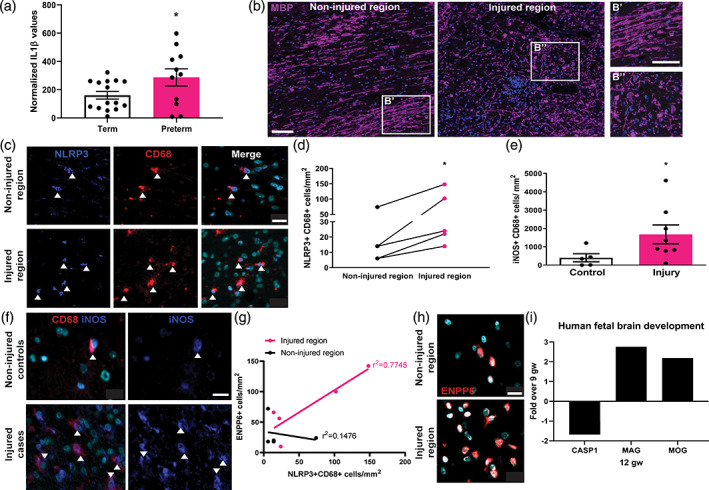
Microglial inflammasome activation in human perinatal brain injury. (a) Normalized values for IL1β protein levels in CSF of term and preterm infants. Two‐tailed Student's *t* test, *p* = .0468. *n* = 11 preterm, 15 term cases. (b) Non‐injured and injured regions of human infant brain stained for MBP (purple) and counterstained with Hoechst (blue). Scale bars, 100 μm. (c) Injured and noninjured regions of human infant brain demonstrating NLRP3 (blue) and CD68 (red) double positive cells (arrows), counterstained with Hoechst (turquoise). Scale bar, 20 μm. (d) Densities of NLRP3+ CD68+ cells/mm^2^ in human developing white matter. Paired *t*‐test, *p* = .0148, *n* = 5 cases. (e) Densities of iNOS+CD68+ cells/mm^2^ in healthy control versus injured human infant brain. Mann–Whitney test, *p* = .0451. *n* = 5 control cases and 8 injured cases. (f) iNOS+ (blue) CD68+ (red) double positive microglia (arrows) in noninjured controls and injured case tissue. Scale bar, 20 μm. (i) NLRP3+CD68+ cells/mm^2^ versus ENPP6+ cells/mm^2^ in injured and non‐injured regions. *n* = 5 cases. (j) Representative images of ENPP6+ cells in injured and non‐injured regions of infant brain. Scale bar, 20 μm. (k) Mean gene expression of CASP1, MAG and MOG in human fetal brain at 12 gestational weeks (gw), fold over levels at 9 gestational weeks

We first asked whether there is differential inflammasome activation in perinatal brain injury compared to healthy development. The NLRP3 inflammasome is composed of NLRP3, a scaffolding protein (ASC), and caspase‐1; complex activation leads to production and activation of interleukin‐1beta (IL1‐β). Thus, we compared protein levels of IL1β in the cerebrospinal fluid (CSF) of infants at high risk of perinatal brain injury (preterm infants with a birthweight <1,500 g)(Back & Rosenberg, 2014; van Tilborg, de Theije, et al., 2018) to those born at term (Supporting Information, Table S1). IL‐1β levels were significantly increased in CSF of preterm versus term infants (Figure 1a). This is consistent with a previous report of increased IL1β detected selectively in damaged white matter of infant postmortem brain, which was most pronounced in preterm infants (Girard, Sebire, & Kadhim, 2010). To determine whether inflammasome activation was associated with microglia, we examined postmortem tissue of infants with recent white matter injury (Table S2; myelin basic protein [MBP] staining indicated in Figure 1b). We found that injured regions had increased densities of inflammasome‐activated (NLRP3+) microglia/macrophages (CD68+) compared to uninjured regions (Figure 1c,d). IL1β induces pro‐inflammatory responses in surrounding cells; accordingly, iNOS+CD68+ cell densities were significantly elevated in white matter of infants with injury compared to control infants who died without a primary neuropathological disease (Figure 1e,f), suggesting a pro‐inflammatory microglia response. NLRP3+CD68+ cell densities were positively correlated with those of immature oligodendrocytes (ENPP6+) in injured regions (Figure 1g,h) but not in noninjured regions (Figure 1g). Conversely, NLRP3+CD68+ cell densities were not positively correlated with those of mature oligodendrocytes (MBP+) (Figure S1), suggesting an association between microglial inflammasome activation and a block in generation of mature myelinating oligodendrocytes. Consistent with this postulate, data‐mining of gene expression profiles of developing human fetal brain (Lindsay et al., 2016) indicated that the gene for caspase‐1 (CASP1: *p* = 1.14 × 10^−12^) is downregulated concomitant with upregulation of myelin genes (MAG: *p* = 2.88 × 10^−7^; MOG: *p* = 1.41 × 10^−6^) (Figure 1i). Altogether, these data suggest that microglial inflammasome activation in perinatal brain injury may impede healthy myelination.

**FIGURE 2:**
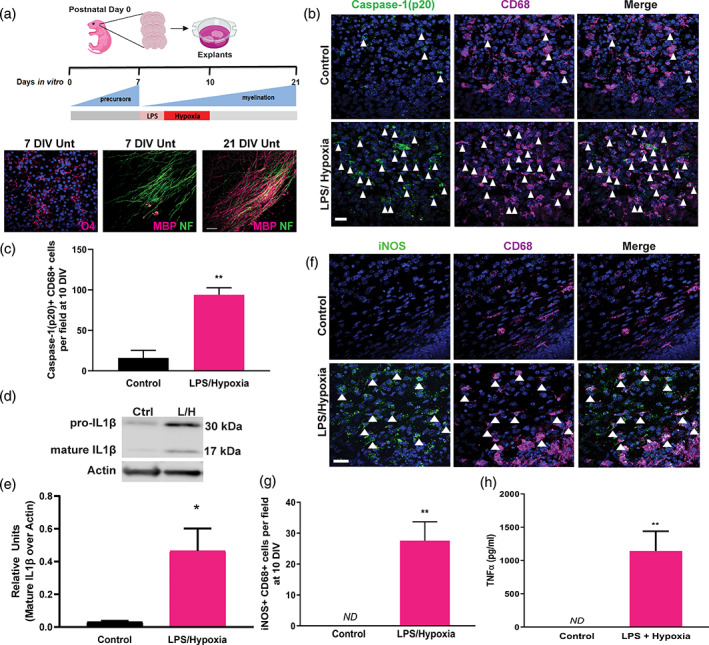
Brain explant model of microglia inflammasome activation. (a) Diagram of mouse forebrain explant model. Explants are isolated from postnatal day 0 pups. At 7 days in vitro (DIV) when oligodendrocyte precursors are abundant, explants are exposed to lipopolysaccharide (LPS) for 2 hr followed by hypoxia (3% O_2_) for 3 days until 10 DIV. Untreated explants (Unt) mirror in vivo oligodendrocyte maturation, at 7 DIV showing abundance of oligodendrocyte precursors (O4+; magenta) and paucity of myelin (MBP+; magenta) on axons (NF+; green), and myelination then being robust by 21 DIV. Scale bar, 25 μm. (b) Control and LPS/Hypoxia exposed explants at 10 DIV immunostained for cleaved caspase‐1 (p20; green) and CD68 (purple), counterstained with Hoechst (blue). Examples of double positive cells are indicated by arrows. Scale bar, 25 μm. (c) Average cleaved caspase‐1 (p20)+CD68+ cells/ field in control and LPS/Hypoxia exposed explants at 10 DIV. Two‐tailed Student's *t* test, *p* = .0018, *n* = 3–4 mice/group. (d) Western blot of control (Ctrl) or LPS/Hypoxia (L/H)‐exposed explants demonstrating pro‐ IL1β (30 kDa) and mature IL1β (17 kDa), against loading control Actin. (e) Quantification of intensity of mature IL1β signal in western blots normalized to actin signal, represented as relative units. Two‐tailed Student's *t* test, *p* = .0340, *n* = 3 mice/group. (f) Control or LPS/Hypoxia‐treated explants at 10 DIV stained for iNOS (green) and CD68 (purple). Examples of double positive cells are indicated by arrows. Scale bar, 25 μm. (g) Mean number of iNOS+ CD68+ cells per field in control and LPS/Hypoxia‐exposed explants. Two‐tailed Student's *t* test, *p* = .0007, *n* = 7 mice/group. *ND* = not detected. (h) Concentration of TNF‐α (pg/ml) secreted from control and LPS/Hypoxia‐exposed explants from 7 to 10 DIV. Two‐tailed Student's *t* test, *p* = 0.0049, *n* = 3 mice/group. *ND* = not detected

To test this postulate, we developed an ex vivo organotypic mouse brain explant model of microglial inflammasome activation in the corpus callosum—a white matter tract commonly affected in injured infants (Figure 2a). Uninjured explants mirrored in vivo development, with abundance of oligodendrocyte precursors (O4+) at 7 days in vitro (DIV) and robust myelination by 21 DIV (Figure 2a). We aimed to activate the inflammasome in explants using stimuli previously associated with both inflammasome activation and developmental white matter injury: a component of the gram‐negative bacterial cell wall (lipopolysaccharide; LPS) and hypoxia (Barnett et al., 2018; Coumans et al., 2003; Dean et al., 2011; Jiang, Stone, Geng, & Ding, 2018; Juliana et al., 2012; van Tilborg et al., 2018). This combination of insults has translational relevance, as gram‐negative bacterial infection is associated with neonatal sepsis, hypomyelination, and development of cerebral palsy (Allard, Brochu, Bergeron, Segura, & Sebire, 2019; Zaidi, Thaver, Ali, & Khan, 2009; Zea‐Vera & Ochoa, 2015), and co‐existing infection and birth asphyxia increases the risk of developmental brain injury compared to either insult alone (Grether & Nelson, 1997; Hayes et al., 2013; Nelson & Grether, 1998; van Tilborg, Achterberg, et al., 2018; Wu et al., 2003). Accordingly, experimental exposure to LPS followed by hypoxia induces white matter injury in the developing brain (Bonestroo, Heijnen, Groenendaal, van Bel, & Nijboer, 2015; Eklind et al., 2001; Kendall et al., 2011; Martinello et al., 2019; Osredkar et al., 2014; Wang et al., 2009). We applied these insults serially, postulating that this would lead to inflammasome activation which requires two successive signals: an initial stimulus to induce NLRP3 and pro‐IL1β expression, and a subsequent stimulus to activate and cleave caspase‐1(“p20”), which in turn cleaves IL1β to its mature form. This paradigm was initiated at seven DIV when oligodendrocyte precursors are most abundant, to mimic the timing of injury in human infants. LPS application for 2 hr followed by hypoxia for 3 days (Figure 2a) induced cleaved‐caspase‐1(p20) primarily in microglia (CD68+), and cleaved‐caspase‐1(p20)+CD68+ cells were significantly increased compared to control at 10 DIV (Figure 2b,c). Inflammasome activation was confirmed by western blotting of explant lysates, which indicated increased pro‐ and mature‐IL1β following LPS and hypoxia exposure (Figure 2d,e). This was associated with increased iNOS+CD68+ microglia (Figure 2f,g) and secretion of TNFα (Figure 2h), suggesting a pro‐inflammatory microglial response.

**FIGURE 3:**
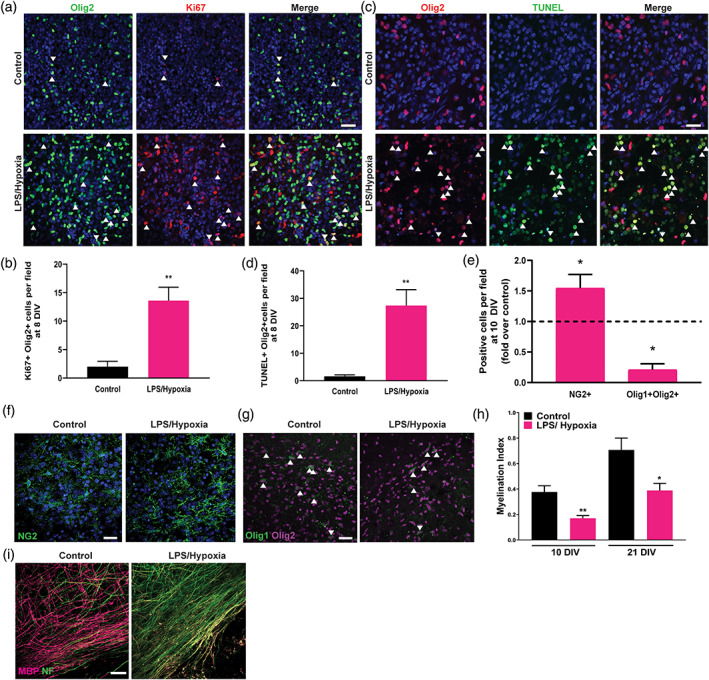
White matter pathology in inflammasome‐activated brain explants. (a) Oligodendrocyte lineage cell (Olig2+; green) proliferation (Ki67+; red) in control and LPS/Hypoxia‐exposed explants at 8 DIV. Examples of double positive cells are indicated by arrows. Scale bar, 25 μm. (b) Mean number of Ki67+ Olig2+ cells per field at 8 DIV in control and LPS/Hypoxia exposed explants. Two‐tailed Student's *t* test, *p* = .0018. *n* = 5 mice/group. (c) Oligodendrocyte lineage cell (Olig2+; red) apoptosis (TUNEL+; green) in control and LPS/Hypoxia‐exposed explants at 8 DIV. Double positive cells indicated by arrows. Scale bars, 10 μm. (d) Mean number of TUNEL+ Olig2+ cells per field at 8 DIV in control and LPS/Hypoxia exposed explants. Two‐tailed Student's *t* test, *p* = .0021. *n* = 5 mice/group. (e) Mean number of NG2+ or cytoplasmic Olig1 + Olig2+ cells per field at 10 DIV in LPS/Hypoxia‐exposed explants normalized to untreated control (dotted line). One sample *t* test, *p* = .0359 (NG2+) and 0.0144 (Olig1 + Olig2+). *n* = 10 and 3 mice/group, respectively. (f) OPCs (NG2+; green) in control and LPS/Hypoxia‐exposed explants at 10 DIV. Scale bar, 25 μm. (g) Differentiating oligodendrocytes identified by cytoplasmic Olig1 (green) and nuclear Olig2 expression (purple) in control and LPS/Hypoxia‐exposed explants at 10 DIV. Scale bar, 25 μm. (h) Myelination index at 10 and 21 DIV in untreated control and LPS/Hypoxia‐exposed explants. Two‐tailed Student's *t* test, *p* = .0076 and 0.0192, respectively, *n* = 3–5 mice/group. (i) Myelination at 21 DIV in explants that were untreated (control) or exposed to LPS/Hypoxia, indicated by MBP (magenta) and NF (green). Scale bar, 20 μm

We then asked whether this microglial inflammasome activation in the explant model was associated with white matter pathology. We found that compared to control, LPS and hypoxia treatment significantly increased oligodendrocyte lineage cell (Olig2+) proliferation (Ki67+) and death (TUNEL+) 1 day later (8 DIV) (Figure 3a–d; Figure S2a,b); increased death was confirmed by live incorporation of a marker of compromised membrane integrity, propidium iodide (Figure S2c,d). This was followed by impaired oligodendrocyte differentiation by 10 DIV, as indicated by increased oligodendrocyte progenitors (NG2+) and decreased differentiating oligodendrocytes (cytoplasmic Olig1+ Olig2+) relative to control (Figure 3e–g); all NG2+ cells in the explant corpus callosum were positive for the oligodendrocyte lineage marker Olig2 (Figure S2e). In contrast, treatment with LPS or hypoxia alone had no impact on oligodendrocyte lineage cell number, proliferation, or differentiation (Figure S2f–h), consistent with previous findings (van Tilborg, Achterberg, et al., 2018). The combination of LPS and hypoxia treatment caused hypomyelination by 10 and 21 DIV, as quantified by area of co‐localization between myelin protein (MBP) and the axonal marker neurofilament (NF), normalized to total NF area (“myelination index”; Figure 3h–i).

**FIGURE 4:**
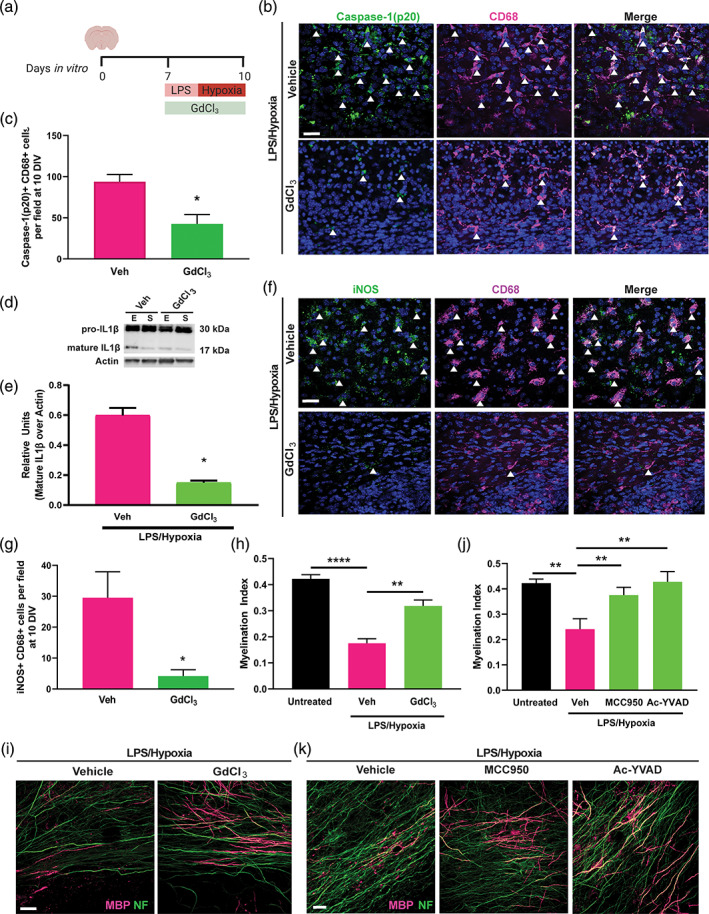
Inflammasome inhibition enhances developmental myelination after injury. (a) Diagram of mouse forebrain explant treatment with LPS/Hypoxia and GdCl_3_ or Vehicle control from 7 to 10 DIV. (b) Cleaved‐caspase‐1 (p20; green) and CD68 (purple) at 10 DIV in LPS/Hypoxia‐exposed explants following treatment with Vehicle or GdCl_3_. Double positive cells indicated by arrows. Scale bar, 25 μm. (c) Mean cleaved caspase‐1 (p20)+CD68+ cells/field at 10 DIV in LPS/Hypoxia‐exposed explants following treatment with Vehicle or GdCl_3_. Two‐tailed Student's *t* test, *p* = .0145. *n* = 3–4 mice/group. (d) Western blot of explant (E) or supernatant (S) lysates following exposure to LPS/Hypoxia and treatment with Vehicle (Veh) or GdCl_3_, indicating pro‐ IL1β (30 kDa) and mature‐IL1β (17 kDa) against loading control β‐actin. (e) Quantification of intensity of mature IL1β signal in western blots normalized to actin signal, represented as relative units. Two‐tailed Student's *t* test, *p* = .0131, *n* = 2 mice/group. (f) LPS/Hypoxia‐exposed explants at 10 DIV, treated with vehicle control or GdCl_3_ during insult, immunostained for iNOS (green) and CD68 (purple) and counterstained with Hoechst (blue). Double positive cells indicated by arrows. Scale bar, 25 μm. (g) Average iNOS+ CD68+ per field in explants at 10 DIV following treatment with vehicle or GdCl_3_ during LPS/Hypoxia exposure. Two‐tailed Student's *t* test, *p* = .0428. *n* = 3 mice/group. (h) Myelination index at 10 DIV for untreated explants and LPS/Hypoxia‐exposed explants treated with Vehicle or GdCl_3_. Two‐tailed Student's *t* test, Untreated versus Vehicle *p* < .0001, Vehicle versus GdCl_3_ *p* = 0.0011. *n* = 5–6 mice/group. (i) MBP (magenta) and NF (green) in LPS/Hypoxia‐exposed explants at 10 DIV following treatment with Vehicle or GdCl_3_. Scale bar, 25 μm. (j) Myelination index at 10 DIV for LPS/Hypoxia‐exposed explants treated with Vehicle, NLRP3 inhibitor MCC950, or caspase‐1 inhibitor Ac‐YVAD. Student's *t* test, Untreated versus Vehicle *p* = .0027, Vehicle versus MCC950 *p* = .0080, Vehicle versus Ac‐YVAD *p* = .0036. *n* = 3–5 mice/group. (k) MBP (magenta) and NF (green) in LPS/Hypoxia‐exposed explants at 10 DIV following treatment with Vehicle, NLRP3 inhibitor MCC950, or caspase‐1 inhibitor Ac‐YVAD. Scale bar, 20 μm

Having developed a model showing microglial inflammasome activation and hypomyelination, we then tested whether there was a causal relationship between the two findings. We applied gadolinium chloride (GdCl_3_) which inhibits the inflammasome and reduces pro‐inflammatory microglia activation following white matter damage (Kim, Kim, & Lee, 2015; Miron et al., 2013; Van Steenwinckel et al., 2019; Xu et al., 2017). GdCl_3_ treatment of explants during LPS and hypoxia exposure (Figure 4a) impaired inflammasome activation in microglia, as indicated by reduced numbers of cleaved caspase‐1(p20)+ CD68+ cells (Figure 4b,c). Western blotting of explant lysates (“E”; Figure 4d) indicated that although pro‐IL1β was not affected by GdCl_3_, mature IL1β was reduced compared to vehicle control (Figure 4e). This was not due to increased secretion of mature IL1β out of the explants as it was largely absent from supernatant lysates (“S”; Figure 4d), likely due to cellular uptake within the explant. iNOS+CD68+ microglia were decreased following GdCl_3_ treatment (Figure 4f,g), whereas total numbers of CD68+ cells were unaltered compared to vehicle control (Figure S3). GdCl_3_‐treated explants demonstrated enhanced myelination by 10 DIV compared to explants exposed to LPS/Hypoxia and vehicle (Figure 4h,i). We confirmed that inhibition of the microglial inflammasome could rescue myelination by application of specific inhibitors of NLRP3 (MCC950) and cleaved caspase‐1 (Ac‐YVAD) and found that myelination indices were significantly increased at 10 DIV compared to LPS/Hypoxia and vehicle‐treated explants (Figure 4j,k). These findings indicate that inflammasome activation in microglia causes hypomyelination.

**FIGURE 5:**
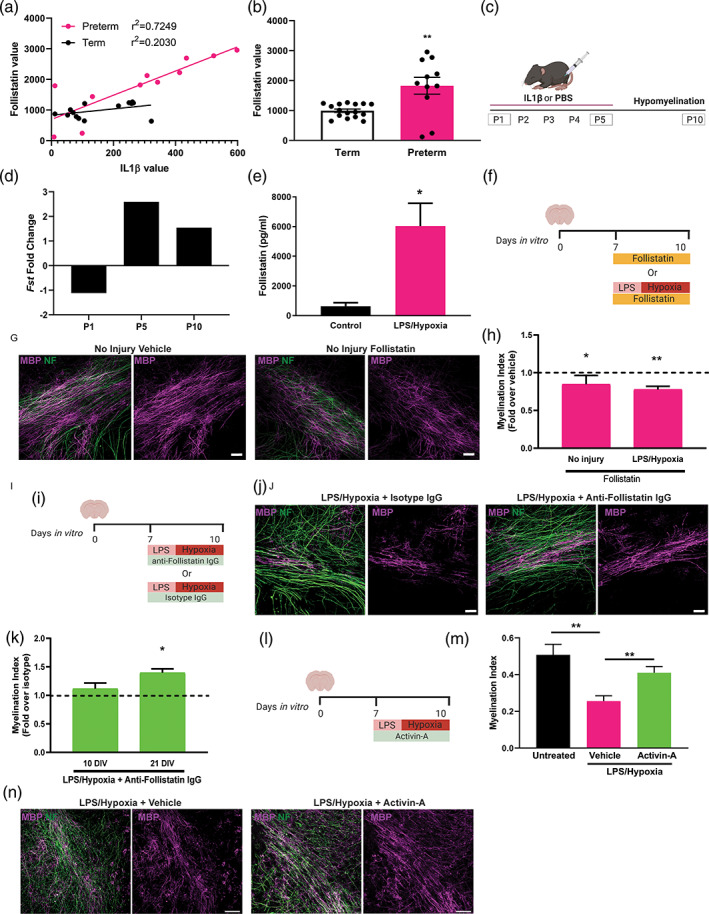
Link between inflammasome activation and inhibition of activin receptor axis in developmental white matter injury. (a) Correlation between IL1β and follistatin normalized protein values in preterm (magenta) and term (black) human infant cerebrospinal fluid (CSF). (b) Normalized value of follistatin protein in term and preterm human CSF. Mann–Whitney test, *p* = .0047. *n* = 11 preterm, 15 term cases. (c) Diagram of in vivo model of perinatal brain injury induced by intraperitoneal injections of IL1β from postnatal day (P) 1 through 5, with hypomyelination observed by P10. (d) Fold change in expression of the follistatin gene (*Fst*) in microglia following IL1β treatment in vivo compared to PBS‐injected control, at P1, P5, and P10. (e) Mean follistatin levels (pg/ml) in conditioned media of 10 DIV explants which were untreated or exposed to LPS/Hypoxia. Student's *t* test, *p* = .0255, *n* = 3 mice per condition. (f) Diagram of treatment of healthy explants or LPS/Hypoxia‐exposed explants with recombinant follistatin or vehicle from 7 to 10 DIV. (g) MBP (purple) and NF (green) in explants with no injury at 10 DIV following treatment with Vehicle or Follistatin. Scale bar, 20 μm. (h) Myelination index at 10 DIV for explants without injury or those exposed to LPS/Hypoxia and treated with Follistatin, normalized to values from vehicle control in each respective condition (dotted line). One sample *t*‐test against value of 1, No injury *p* = .0188, LPS/Hypoxia *p* = .0030. *n* = 3 mice/group. (i) Diagram of treatment of explants with anti‐follistatin IgG or isotype IgG during LPS/Hypoxia exposure from 7 to 10 DIV. (j) MBP (purple) and NF (green) in LPS/Hypoxia‐exposed explants at 10 DIV following treatment with Anti‐Follistatin IgG or control Isotype IgG. Scale bar, 20 μm. (k) Myelination index at 10 and 21 DIV in LPS/Hypoxia‐exposed explants treated with anti‐Follistatin IgG, normalized to values from isotype IgG control at each time point (dotted line). One sample *t*‐test against value of 1, *p* = .0276. *n* = 3 mice/group. (l) Diagram of treatment of explants with recombinant activin‐A or vehicle control during LPS/Hypoxia exposure from 7 to 10 DIV. (m) Myelination index at 21 DIV of untreated explants or those exposed to LPS/Hypoxia and treated with either vehicle or activin‐A from 7 to 10 DIV. Two‐tailed Student's *t* test, Untreated versus Vehicle *p* = .0014, Vehicle versus Activin‐A *p* = .0063. *n* = 5–10 mice/group. (n) LPS/Hypoxia‐treated explants treated with vehicle control or activin‐A and stained for MBP (purple) and neurofilament (green) at 21 DIV. Scale bar, 75 μm

We next sought to determine how microglial inflammasome activation inhibits myelination. We previously identified five proteins which are highly significantly altered in preterm versus term CSF (C5a, IL9, FasL, follistatin, and SPP1) (Boardman et al., 2018), and asked whether these were correlated with levels of IL1β. Whereas in preterm infants, there was poor correlation between IL1β and C5a (*r* ^2^ = 0.2863), IL9 (*r* ^2^ = 0.04792), FasL (*r* ^2^ = 0.01415), and SPP1 (*r* ^2^ = 0.4192) (Figure S4), we found a robust correlation with levels of follistatin (*r* ^2^ = 0.7249) (Figure 5a); there was, however, poor correlation between IL1β and follistatin in term controls (*r* ^2^ = 0.2030; Figure 5a). In addition, follistatin protein values were significantly increased in preterm versus term infant CSF (Figure 5b), associating it with increased risk of brain injury. We then asked whether IL1β can induce follistatin expression in microglia. We examined published transcriptomes of microglia from an in vivo model which mimics downstream inflammasome activation by injection of IL1β in mice from postnatal day (P)1 through 5, inducing hypomyelination by P10 (Figure 5c) (Krishnan et al., 2017). We found that IL1β exposure in vivo increased follistatin (*Fst*) levels in microglia by P5 and P10 (Figure 5d). In our explant model, we found increased follistatin protein levels in the conditioned media following treatment with LPS and hypoxia compared to control at 10 DIV (Figure 5e), a time when we observed increased inflammasome activation in microglia. Given that follistatin specifically inhibits activin‐A from binding its receptors, and we recently showed that activin‐A signaling is required for myelination in development (Dillenburg et al., 2018), we postulated that increased follistatin could impede myelination. Indeed, treating healthy explants with recombinant follistatin from 7 to 10 DIV (Figure 5f) was sufficient to significantly reduce the myelination index at 10 DIV (Figure 5g,h). Additionally, hypomyelination at 10 DIV following LPS and hypoxia exposure was worsened with follistatin treatment (Figure 5h). We then asked whether we could alleviate the detrimental effect of follistatin on myelination in injured explants, using two different approaches. First, we blocked follistatin in injured explants by applying a neutralizing antibody during LPS and hypoxia exposure (Figure 5i). We found that blocking follistatin enhanced myelination compared to LPS and hypoxia‐exposed explants treated with isotype IgG control (Figure 5j,k), which reached significance by 21 DIV. Second, we asked whether supplementing injured explants with exogenous activin‐A (Figure 5l) could overwhelm the follistatin to allow myelination to take place. We found that activin‐A treatment of LPS and hypoxia‐exposed explants enhanced myelination index by 21 DIV (Figure 5m,n). These findings reveal an unexpected link between microglial inflammasome activation and activin‐A signaling.

## 4 DISCUSSION

By analysis of human infant brain tissue and cerebral spinal fluid combined with the use of a novel explant model of perinatal brain injury, we reveal that developmental hypomyelination is driven by microglial activation of the NLRP3 inflammasome (see Table of Contents Image). This builds on previous work demonstrating that exogenous application of the inflammasome product IL1β is sufficient to induce hypomyelination in experimental models (Favrais et al., 2011; Krishnan et al., 2017; Mairesse et al., 2019), and that human genetic mutations in *NLRP3* causing excess IL1β production are associated with neonatal periventricular white matter lesions (Goldbach‐Mansky et al., 2006). We now demonstrate that inflammasome inhibition reinstates myelination in developing white matter, a strategy previously shown to dampen injury to neurons in development (Chen, Xu, & Yuan, 2018; Girard et al., 2012; Leitner et al., 2014) and to white matter in adulthood (Cantuti‐Castelvetri et al., 2018; Malhotra et al., 2020).

Microglial inflammasome activation in infants may occur due to exposure to two insults within a short time window to provide priming and activation signals. Accordingly, compound insults in infants are associated with severe white matter injury and increased risk of developing cerebral palsy (Barnett et al., 2018; Grether & Nelson, 1997; Hayes et al., 2013; Nelson & Grether, 1998; Wu et al., 2003). Furthermore, clinical contexts often associated with perinatal brain injury—systemic infection, encephalopathy and ischemic stroke—would be predicted to provide signals which induce inflammasome activation, such as pathogen‐associated molecular patterns, hypoxia, reactive oxygen and nitrogen species, and calcium fluxes (Hagberg et al., 2015; Yang, Wang, Kouadir, Song, & Shi, 2019). This postulate was affirmed by our demonstration that sequential exposure to the gram‐negative *E. coli* cell wall component LPS and hypoxia was sufficient to induce inflammasome activation in microglia in developing white matter. Additionally, recent work demonstrated that although exposure of developing mouse brain explants to LPS alone primes the inflammasome, it does not lead to inflammasome activation nor mature IL1β production (Hoyle et al., 2020), presumably missing the second stimuli for full inflammasome complex activation.

Consistent with this finding, we observed that a combination of LPS and hypoxia was needed to significantly impact the oligodendrocyte lineage, leading to hypomyelination. This is of translational relevance, as gram‐negative bacterial infection contributes to hypomyelination and development of cerebral palsy, and co‐existing infection and birth asphyxia significantly increase the risk of perinatal brain injury and cerebral palsy compared to either insult in isolation (Allard et al., 2019; Grether & Nelson, 1997; Hayes et al., 2013; Nelson & Grether, 1998; Wu et al., 2003). Previous experimental work in various models (rodent, sheep, piglet) have shown that LPS sensitizes the brain to subsequent hypoxia to exacerbate developmental brain injury (Bonestroo et al., 2015; Eklind et al., 2001; Kendall et al., 2011; Martinello et al., 2019; Osredkar et al., 2014; Wang et al., 2009). Altogether, this demonstrates that the combination of LPS and hypoxia induces microglial inflammasome activation, which drives white matter pathology similar to that observed in human infants with perinatal brain injury.

We reveal that microglial inflammasome activation induces white matter pathology via regulation of the activin receptor axis. We found a correlation between IL1β and increased levels of the endogenous activin‐A inhibitor follistatin in the context of human and mouse developing white matter injury. IL1β is known to modulate follistatin levels and activin receptor signaling in skin and the reproductive system (Arai et al., 2011; Bilezikjian et al., 1998), and here we implicate the crosstalk between these pathways in microglia‐driven hypomyelination. We complement our previous work showing that activin receptor signaling is required for myelination in development (Dillenburg et al., 2018), by now demonstrating that increased follistatin levels following perinatal brain injury impair myelination. Poor myelination was overcome by neutralizing follistatin or supplementation with exogenous activin‐A. This leads to the postulate that reinstating activin receptor signaling could represent a promising therapeutic strategy to promote healthy white matter development in injured infants.

Overall, our findings identify a molecular link between dysregulated microglia activation and hypomyelination in perinatal brain injury, and point to novel therapeutic strategies to support white matter health in injured infants centered on inflammasome inhibition and/or reinstatement of activin receptor signaling.

## Supporting information


**Appendix S1**: Supporting Information

## Data Availability

All data is available from the corresponding author upon request.
