# Supplementary material for: Microglial inflammasome activation drives developmental white matter injury
Source: Glia. 2021 Jan 8;69(5):1268–80. doi: 10.1002/glia.23963 (PMC8607465; doi:10.1002/glia.23963)
Supplement: Supplementary file 1 — Appendix S1: Supporting Information [file GLIA-69-1268-s001.docx]

**Glia: Original Research Article**

**Microglial inflammasome activation drives developmental white matter injury**

**Appendix**

Tables 1 & 2

Supplemental Figures 1-4

**Supplementary Table 1. Cerebrospinal fluid case information**

| **Type** | **Case** | **Gestational age at birth**  **(weeks)** | **Age at lumbar puncture**  **(weeks)** | **Birth**  **weight**  **(g)** |
| --- | --- | --- | --- | --- |
| **Term** | 1 | 40.71 | 40.71 | 2770 |
|  | 2 | 40.14 | 40.28 | 3130 |
|  | 3 | 38 | 38 | 4730 |
|  | 4 | 36.85 | 37.14 | 3480 |
|  | 5 | 37.42 | 38.28 | 3410 |
|  | 6 | 41.71 | 41.85 | 4450 |
|  | 7 | 41.57 | 42.28 | 3505 |
|  | 8 | 40.57 | 41.57 | 3340 |
|  | 9 | 41.57 | 42 | 3840 |
|  | 10 | 41.42 | 41.5 | 3840 |
|  | 11 | 41.42 | 41.42 | 2840 |
|  | 12 | 38.28 | 38.28 | 2212 |
|  | 13 | 40.71 | 41 | 3210 |
|  | 14 | 42 | 42 | 3390 |
|  | 15 | 37 | 37.28 | 3950 |
| **Preterm** | 1 | 29.28 | 30.57 | 1440 |
|  | 2 | 26 | 27.42 | 900 |
|  | 3 | 26.85 | 27.71 | 1310 |
|  | 4 | 27.85 | 28.42 | 1005 |
|  | 5 | 28.28 | 30 | 730 |
|  | 6 | 24.57 | 26.28 | 810 |
|  | 7 | 23.42 | 23.57 | 500 |
|  | 8 | 31.71 | 32 | 1520 |
|  | 9 | 25.71 | 29.57 | 935 |
|  | 10 | 28.28 | 32 | 1040 |
|  | 11 | 27.71 | 28.71 | 1100 |

**Supplementary Table 2. Post-mortem tissue case information**

| **Type** | **Case** | **Gestation**  **(weeks)** | **Survival** | **Diagnosis** |
| --- | --- | --- | --- | --- |
| **Injury** | 1 | 33 | 0d | Intrauterine growth restriction, hypoxia |
|  | 2 | 41 | 4d | Periventricular leukomalacia, hypoxia |
|  | 3 | 24 | 0d | Hypoxia |
|  | 4 | 27 | 6d | Subependymal and intraventricular haemorrhage |
|  | 5 | 38 | 0d | Hypoxia |
|  | 6 | 27 | 4wk | Periventricular leukomalacia |
|  | 7 | 27 | 5wk | Periventricular leukomalacia |
|  | 8 | 30 | 0d | Hypoxic Ischemic Encephalopathy |
|  | 9 | 30 | 0d | Periventricular leukomalacia |
|  | 10 | 32 | 0d | Hypoxic Ischemic Encephalopathy and Periventricular Leukomalacia |
|  | 11 | 37 | 5d | Hypoxic Ischemic Encephalopathy |
|  | 12 | 35 | 6d | Hypoxic Ischemic Encephalopathy |
|  | 13 | 36 | 5d | Hypoxic Ischemic Encephalopathy |
| **Control** | 1 | 28 | 0d | Stillbirth |
|  | 2 | 36 | 0d | Diaphragmatic hernia |
|  | 3 | 37 | 3d | Diaphragmatic hernia |
|  | 4 | 37 | 2d | Stillbirth |
|  | 5 | 37 | 2d | Stillbirth |

**Supplementary Figure 1: NLRP3+ CD68+ cells are not positively correlated with MBP+ cells in human infant brain**

**
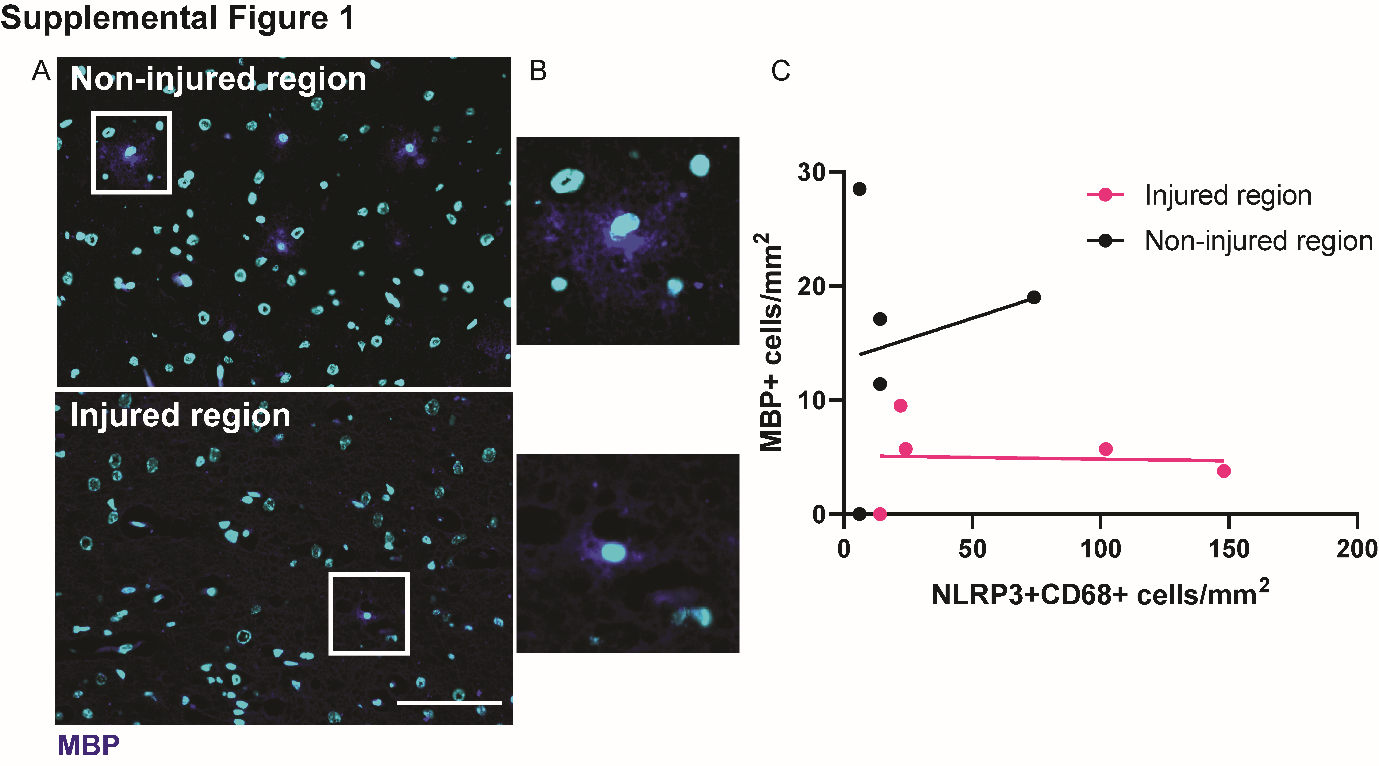
**

A) MBP+ cells (blue) in non-injured and injured regions of human infant brain, Hoechst counterstain in turquoise. Scale bar, 20 µm.

B) Magnified view of MBP+ cell from inset in (A).

C) Correlation of density of NLRP3+CD68+ cells to MBP+ cells per mm^2^ in injured regions (magenta) and non-injured regions (black) of human infant brain.

**Supplementary Figure 2: Assessment of oligodendrocyte lineage cell responses in explants**


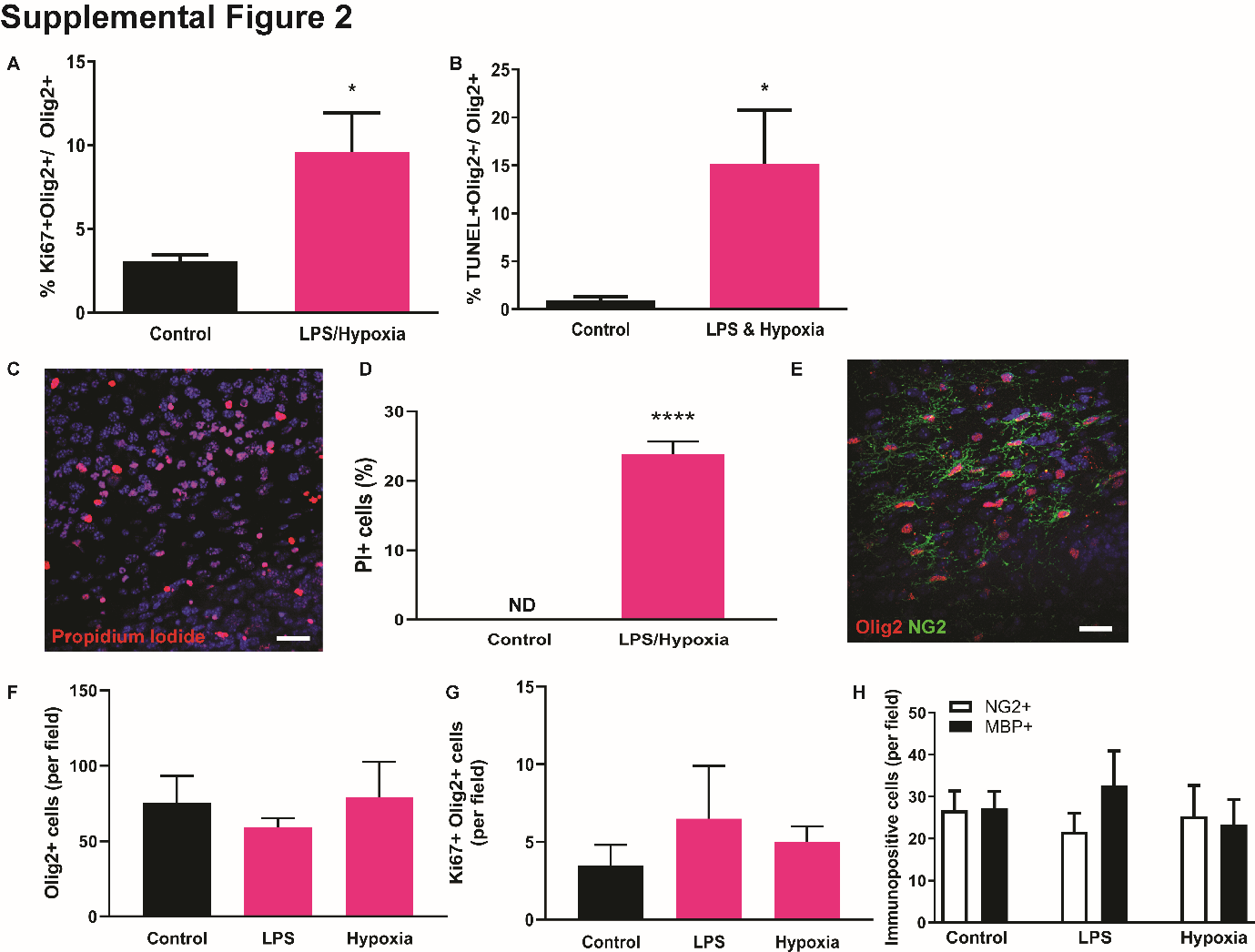


A) Mean percentage of oligodendrocyte lineage cells (Olig2+) which are proliferating (Ki67+) in control and LPS/Hypoxia-exposed explants at 8 DIV. 2-tailed Student’s t-test, *P*=0.05, n=3 mice/group.

B) Mean percentage of oligodendrocyte lineage cells (Olig2+) which are apoptotic (TUNEL+) in control and LPS/Hypoxia-exposed explants at 8 DIV. 2-tailed Student’s t-test, P=0.0347, n=5 mice/group.

C) Propidium iodide labelling of dying cells (red), counterstained with Hoechst (blue). Scale bar, 25 µm.

D) Percentage of cells which are propidium iodide (PI) positive in control and LPS/Hypoxia-exposed explants at 8 DIV. ND=not detected. 2-tailed Student’s t-test, *P*<0.0001. n=5 mice/group.

E) Representative image of colocalization of Olig2 (red) and NG2 (green) in explants. Scale bar, 25 µm.

F) Mean number of oligodendrocyte lineage cells (Olig2+) cells per field at 8 DIV in control explants or those exposed to LPS or hypoxia alone. n=3-4 mice/group.

G) Mean number of oligodendrocyte lineage cells (Olig2+) per field which are proliferating (Ki67+) at 8 DIV in control of explants exposed to LPS or hypoxia alone. n=2-4 mice/group.

H) Mean number of NG2+ or MBP+ cells per field at 10 DIV in control or explants exposed to LPS or hypoxia alone. n=3-4 mice/group.

**Supplementary Figure 3: CD68+ cells are unchanged in GdCl_3_-treated explants**

**
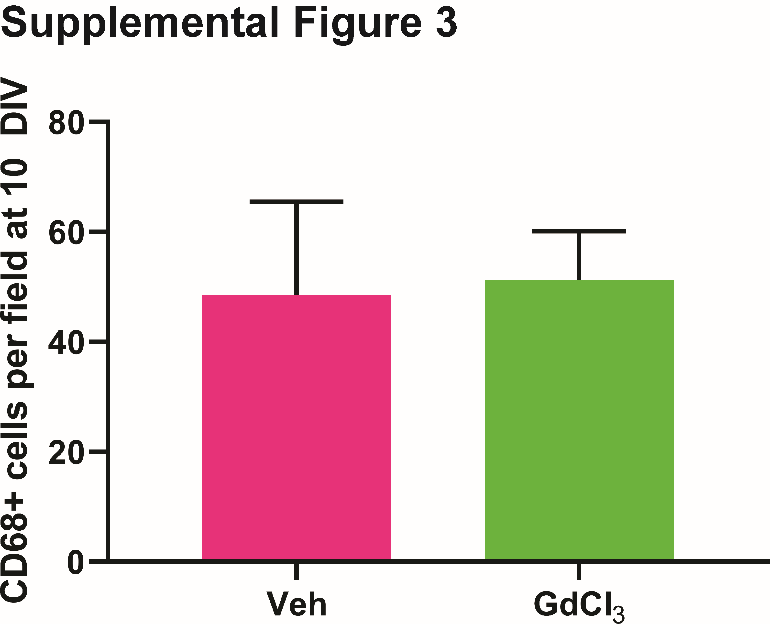
**

Mean number of CD68+ cells per field at 10 DIV in LPS/Hypoxia-exposed explants treated with either Vehicle control (Veh) or GdCl_3_ from 7-10DIV. n=3 mice/group.

**Supplementary Figure 4: Correlation between IL1β and proteins enriched in preterm cerebrospinal fluid**


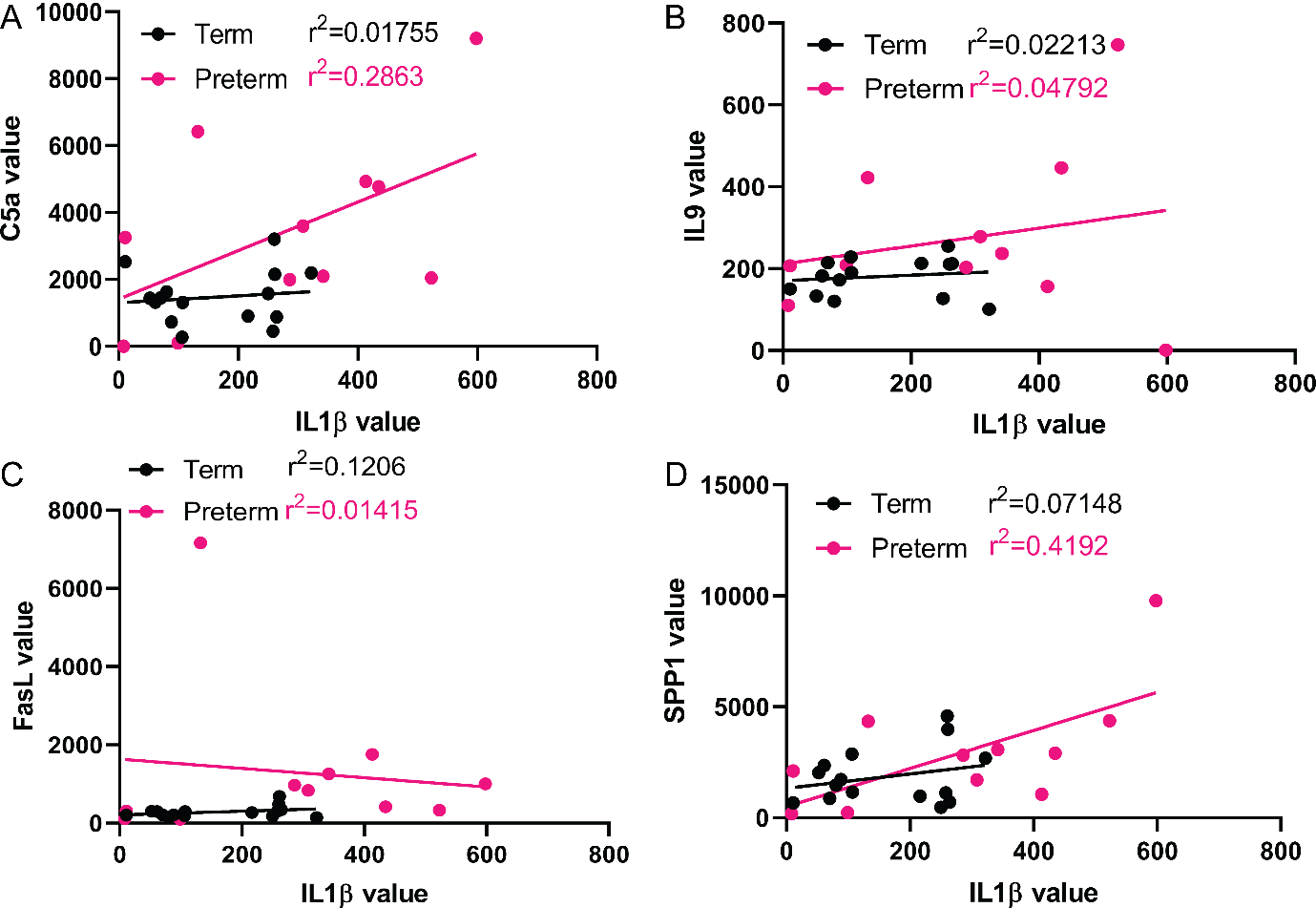


Correlations between protein values of IL1β and those of proteins previously found to be enriched in preterm infant cerebrospinal fluid compared to term infant controls: C5a (A), IL9 (B), FasL (C) and SPP1 (D). Term infant values are indicated in black whereas preterm infant values are indicated in magenta.
